# Risk factors for avian influenza virus contamination of live poultry markets in Zhejiang, China during the 2015–2016 human influenza season

**DOI:** 10.1038/srep42722

**Published:** 2017-03-03

**Authors:** Xiaoxiao Wang, Qimei Wang, Wei Cheng, Zhao Yu, Feng Ling, Haiyan Mao, Enfu Chen

**Affiliations:** 1 Zhejiang Provincial Centre for Disease Control and Prevention, Hangzhou, People’s Republic of China; 2 Ningbo University, Ningbo, People’s Republic of China

## Abstract

Live bird markets (LBMs), being a potential source of avian influenza virus, require effective environmental surveillance management. In our study, a total of 2865 environmental samples were collected from 292 LBMs during the 2015–2016 human influenza season from 10 cities in Zhejiang province, China. The samples were tested by real-time quantitative polymerase chain reaction (RT-PCR). Field investigations were carried out to investigate probable risk factors. Of the environmental samples, 1519 (53.0%) were contaminated by A subtype. The highest prevalence of the H9 subtype was 30.2%, and the frequencies of the H5 and H7 subtype were 9.3% and 17.3%, respectively. Hangzhou and Jinhua cities were contaminated more seriously than the others. The prevalence of H5/H7/H9 in drinking water samples was highest, at 50.9%, and chopping board swabs ranked second, at 49.3%. Duration of sales per day, types of live poultry, LBM location and the number of live poultry were the main risk factors for environmental contamination, according to logistic regression analysis. In conclusion, LBMs in Zhejiang were contaminated by avian influenza. Our study has provided clues for avian influenza prevention and control during the human influenza season, especially in areas where LBMs are not closed.

Influenza A virus is a single-stranded negative-sense RNA virus, which is composed of eight gene segments^1^. To date, 18 H subtypes and 11 N subtypes have been identified, according to the properties of hemagglutinin (HA) and neuraminidase (NA), forming the vast reservoir of influenza virus^2^. It has been estimated that influenza A virus causes about 3–5 million severe cases and 25,000 to 50,000 deaths every year worldwide^3^.

During the twentieth century, influenza epidemics occurred four times. Other than the Spanish influenza pandemic from 1918 to 1920^4^, all the epidemics, Asian flu (H2N2) in 1957, Hong-Kong flu (H3N2) in 1968, and Russian flu (H1N1) in 1977, occurred in Asian and originated in China^5^. In 1997, 18 humans were infected by H5N1 in Hong Kong and six of them died, which was the first indication that the influenza virus could jump the species barrier and thus pose an enormous threat to people^6^. Over recent decades, human infections have been caused by various subtypes of avian influenza virus, including H10N8, H5N6, and H9N2. Most of them were first reported in China. In early 2013, a novel avian-origin reassortant influenza A (H7N9) virus emerged in Shanghai and Anhui, and then rapidly swept across other provinces of China^7^. As of 29th September, 2016, 755 cases of avian influenza A (H7N9), which causes high mortality in humans (41.9%), had been identified in 19 provinces of mainland China^8^. However, it has low or even no pathogenicity in other animals^9^. Therefore, H7N9 virus may be more of a challenge than highly pathogenic H5N1 avian influenza at some levels.

Surveillance is the most important preventive measure for assessment of the current contamination situation and timely discovery of virus variation, so that effective measures may be implemented to control the disease. Some researchers^10^,^11^ have reported findings from environmental surveillance. Horm^10^ found that the samples collected from farms had a higher H5N1 positive rate than those from ponds. Another study^11^ observed that the H7N9 positive rate in live poultry markets (LBMs) was higher than those on farms and in slaughter houses. Poor management, such as the absence of regular rest days to clean and disinfect the premises, causes severe hygiene problems and the spread of viruses in LBMs^12^. Of the laboratory-confirmed cases of avian influenza A (H7N9), 75% had a history of poultry exposure^13^. In addition to H7N9, LBMs are also the major source of human infections with other avian influenza subtypes, such as H10N8, H5N6, H5N1 and H9N2^14^,^15^,^16^. Zhejiang, situated in the core area of Yangtze River Delta, is an area of high incidence of avian influenza^17^. Being a province with a powerful economy, it has close trade links, including live poultry transactions, with other provinces. Thus, avian influenza viruses are easily spread across Zhejiang^18^. Shutting down the LBMs may be an ideal control measure. However, it is traditional for consumers to purchase live birds at local markets and slaughter them at home. Therefore, it is important to manage LBMs effectively with the use of environmental surveillance.

Several surveillance studies^19^,^20^ have provided evidence of avian influenza contamination; however, most of them have focused only on one subtype and they have rarely systematically explored the positive rate of various subtypes in LBMs. In this study, we aimed to identify the prevalence of subtype A, H5, H7 and H9 in LBMs by collecting environmental samples twice monthly during the human influenza season. We also investigated LBM-related information to evaluate the risk factors for environmental contamination. This research was intended to provide data and scientific evidence for risk assessment and disease prevention.

## Results

### General information

Among the environmental samples, 1519 (53.0%) samples from 240 (82.2%) LBMs were contaminated by subtype A avian influenza; 866 (30.2%) of the environmental samples from 178 (61.0%) LBMs were H9 positive, which was a higher frequency than for the H5 and H7 subtypes, whose prevalence was 7.9% (226) and 17.3% (497), respectively. In addition, 1138 (39.7%) environmental samples from 213 (72.9%) LBMs were contaminated by H5/H7/H9 subtypes.

### Regional distribution of avian influenza in environmental samples

**Figure 1:**
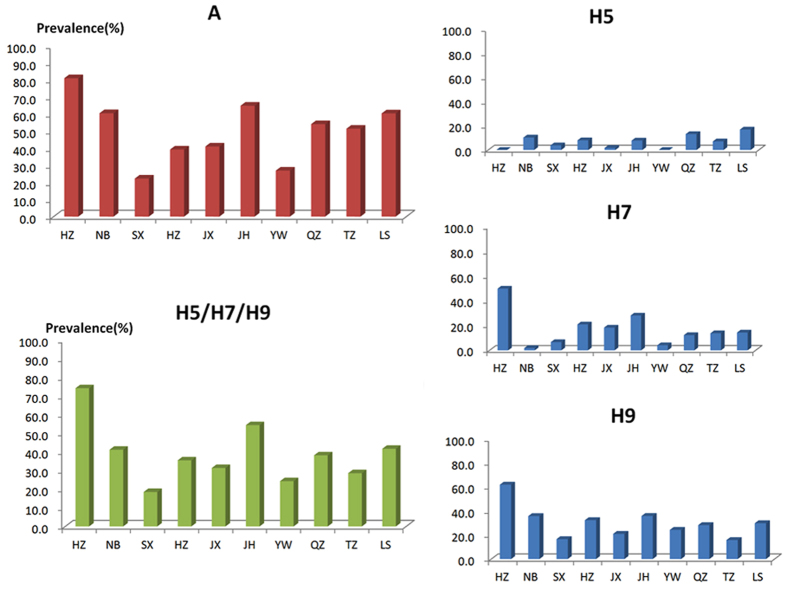
Prevalence of avian influenza subtypes in environmental samples from 10 cities.

Avian influenza A and H5/H7/H9 subtypes had a similar regional distribution in Zhejiang province. In general, the prevalence of the subtypes was ranked as follows: H9 > H7 > H5, except that Ningbo, Quzhou and Lishui had a slightly higher prevalence of H5 than H7 (Table 1). Hangzhou and Jinhua were contaminated more seriously than the other districts. Shaoxing and Yiwu had relatively lower positive rates of avian influenza (Fig. 1).

**Figure 2:**
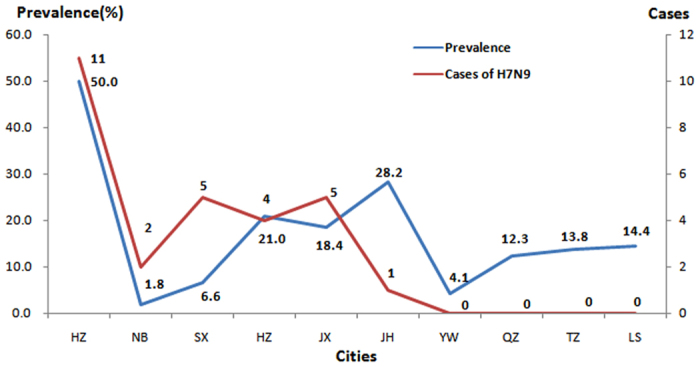
Prevalence of H7 subtype in LBMs and the human cases of H7N9 in 10 cities.

In terms of regional distribution, a positive correlation (*r* = 0.697, *P* = 0.025) was found between the H7 positive rate and the number of H7N9 cases. Hangzhou had a higher H7 positive rate and more cases of H7N9 than the other districts. However, it was noteworthy that Jinhua had the highest positive rate in environmental samples but only one case of H7N9 was identified at the same time (Fig. 2).

### Temporal distribution of avian influenza in environmental samples

**Figure 3:**
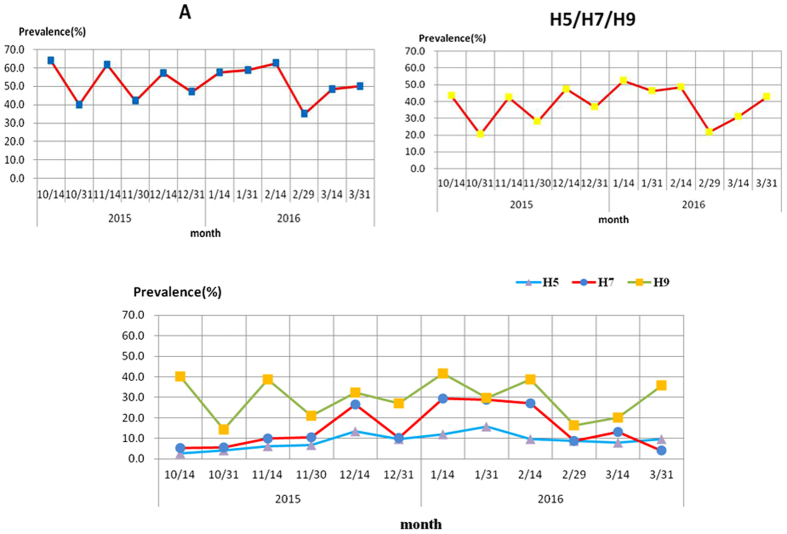
Prevalence of avian influenza subtypes in environmental samples during the 2015–2016 human influenza season.

As shown in Fig. 3, the prevalence of A and H5/H7/H9 subtypes had a similar trend, while H5 and H7 differed. The prevalence of influenza A subtypes remained at a high level (between 40.0% and 64.0%) during the human influenza season, while the prevalence of H5, H7 and H9 fluctuated between 2.6% to 15.6%, 4.0% to 29.4% and 14.4% to 41.6%, respectively. The prevalence of subtype A peaked in the middle of October; the highest frequencies of H5 and H7 detection, however, were observed in January.

### Detection of avian influenza in different sample types

The chi-squared test indicated that the distribution of avian influenza subtypes in all types of sample were statistically different (*P* < 0.001). Drinking water samples had the highest positive rate (73.2%) of the A subtypes, while fecal dropping swabs had the lowest (44.2%). The H5 subtype was mainly found in poultry drinking water samples (16.5%) and in sewage samples (16.5%). Except for other poultry-related samples, the highest H7 positive rate (22.2%) occurred in the swabs taken from chopping boards. For the H9 subtype, contamination of drinking water samples (39.7%) was at higher level than with the other four types of sample; this was also the case for A subtype. In addition, 114 (50.9%) poultry drinking water samples were positive for H5/H7/H9 subtypes. Fecal dropping swabs had a lower positive rate than other sample types (Table 2).

### Risk factors for contamination of the LBM environment

Univariate analysis showed that the factors associated with the prevalence of subtype A virus were the duration of sales per day (*Z* = −2.075, *P* = 0.038) and the sanitary conditions of the LBM (*x*^2^ = 7.448, *P* = 0.024). The more time available to sell live poultry per day, the worse sanitary conditions were found to be, which was associated with a higher prevalence of A subtype in the LBMs. Sales days of less than 10 hours (*Z* = −2.677, *P* = 0.007), a large market trading area (*x*^2^ = 11.983, *P* = 0.003), multiple types of live poultry (*Z* = −2.305, *P* = 0.021) and disinfection and cleaning (*Z* = −2.963, *P* = 0.003) were the risk factors associated with H5 prevalence. However, LBMs located in urban areas (*Z* = −2.727, *P* = 0.006) with better sanitary conditions (*x*^2^ = 6.139, *P* = 0.046) had lower H7 prevalence (Table 3).

As shown in Table 4, logistic regression analysis revealed that the positive rate of subtype A had three risk factors: longer duration of sales per day (odds ratio (*OR*) = 1.141, 95% credibility interval (*CI*) = 1.011–1.288), selling two or more types of live poultry (*OR* = 2.210, 95%*CI* = 1.026–4.758) and LBMs in rural areas (*OR* = 2.790, 95%*CI* = 1.374–5.667). In addition, three risk factors affected the prevalence of H7 subtype: longer duration of sales per day (*OR* = 1.153, 95%*CI* = 1.045–1.273), increasing the density of live poultry in the LBM (*OR* = 1.002, 95%*CI* = 1.000–1.004) and LBMs in rural areas (*OR* = 2.031, 95%*CI* = 1.115–3.699).

## Discussion

According to previous studies^13^,^21^,^22^, LBMs are a potential source of human infections with avian influenza. Therefore, it was not surprising that more than 80% of the LBMs, including over half the samples, were contaminated by influenza A in our study. This finding reinforces the fact that the environmental hygiene of LBMs in Zhejiang province needs to be improved substantially. In our study, the H9 subtype had a higher prevalence than the H5 and H7 subtypes, which indicates that H9 is the predominant subtype in LBMs in Zhejiang province during the human influenza season. Wu *et al*.^23^ found, similarly, that H9 was the primary subtype, accounting for 31.8% of viruses isolated during January 2013 to December 2014 in Zhejiang. To date, although human infections with the H9 subtype have rarely been reported in China^24^, there is also a high risk of human infection with avian influenza caused by the H9 subtype. Another study, from Shantou city, China, also showed that the H9 subtype was the most prevalent, while the H5 subtype was rare^25^. However, the prevalence of the H9 subtype (8.1%) isolated from LBMs in Korea was far lower than that (32.2%) in our study^26^.

It is well known that Zhejiang province has a relatively high number of human infections with H7N9, especially Hangzhou. During the past four waves of the outbreak, Hangzhou had the largest number of cases in Zhejiang. In our study, the H7 positive rate was significantly higher in Hangzhou than in the other districts. This was the direct evidence found in our study to illustrate that Hangzhou is a high risk area for avian influenza and human infection with H7N9 virus. Hangzhou, as a provincial capital, should be set up as a good example for other districts in Zhejiang. However, this may be difficult for metropolitan areas because Hangzhou is also the live poultry distribution center in Zhejiang and live poultry derived from different districts are gathered in the town. In addition, we found that the prevalence of subtype A peaked in the middle of October. However, the frequencies of H5 and H7 detection remained at a relatively low level at this time. Undoubtedly, other subtypes such as H3 and H10, rather than H5 and H7, can increase the prevalence of influenza A^23^. Even so, H5, H7 and H9 remain the prevalent subtypes in LBMs in Zhejiang.

Other studies^19^,^27^ have found that the slaughter zone and sale zone in LBMs were the most seriously contaminated areas. In our study, we found that the drinking water samples had the highest prevalence of A, H5, and H9 subtypes. This is not unexpected, because drinking water shared by different live birds may preserve secretions such as saliva which are loaded with many influenza viruses and serve as the viral vector^19^. In addition, during the process of slaughter, droplets containing viral particles may be produced and spread within the narrow and poorly ventilated space^27^, so that the A, H5, H7 and H9 subtypes were detected in swabs taken from chopping boards and in sewage samples. Kang *et al*.^20^ reported a similar result: they found many H7N9-positive samples in chopping tools. In contrast, lower detection rates for A, H5, H7 and H9 were found in the fecal dropping swabs. One study^19^ showed that the way cages were arranged was an important factor in contamination by influenza virus. Vertical stacking was able to control the distribution of fecal matter effectively, which could reduce the rate of contamination in LBMs. In general, tools related to poultry should be considered as the main objects to be cleaned and disinfected.

Univariate and logistic regression analysis demonstrated that there were several factors associated with environmental contamination, including the duration of sales per day, the market trading area, types of live poultry, LBM location, rest days, disinfection and cleaning, LBM hygiene and the number of live poultry. The reasons for this may be as follows: First, a longer duration of sales increases the likelihood of virus spread. Fournie *et al*.^28^ also considered that opening the LBMs all day long was in favor of virus transmission. The large number of influenza virus particles in the environment would be transmitted to other live poultry and species by contact with contaminative fomites^29^,^30^,^31^. Second, large LBMs such as wholesale markets are generally loaded with different types and high densities of live poultry. Several studies^28^,^32^,^33^,^34^ have shown that a large quantity and high densities of live poultry may increase virus activity and thus strengthen the risk of infection, which would be beneficial to dissemination and genetic reassortment^35^,^36^. It is noteworthy that traders often do not clearly divide poultry holding, slaughtering and selling into different zones, which would facilitate cross-contamination, especially in the LBMs with many types of live poultry^19^,^37^. Third, the terrible sanitary conditions and lack of management in certain LBMs aggravates the risk of contamination^38^. Fourth, in contrast to previous studies^12^,^39^,^40^ which have indicated that regular rest days, disinfection and cleaning minimize the contaminants in LBMs, our study found that regular rest days, disinfection and cleaning were risk factors associated with H5 subtype contamination. The contradiction may derive from: 1) recall bias and reporting bias, 2) more serious contamination may have driven the administration section of the LBMs to disinfect and clean more frequently. Further surveys and cohort studies are needed to investigate the direction of causality between regular rest days, disinfection, cleaning and H5 prevalence. Other studies have shown that two groups among poultry-related workers, including rural people and those with lower educational level, were less likely to follow the rules strictly owing to their lack of knowledge related to avian influenza virus^38^,^41^. Thus, it is critical to promote measures aimed to improve knowledge of avian influenza.

According to the evidence from our study, we suggest placing special emphasis on daily management of LBMs, especially in areas where LBMs are not closed. As the LBMs are closed in downtown areas of Zhejiang province, risks for human infection with avian influenza transfer to other areas with LBMs. As our data show, shortening sales duration, setting a limit to live poultry quantity and types, or appropriate partition design to divide various kinds of poultry, and scientific management of rural LBMs are positive approaches to reducing the risk. Other studies have also supplied measures, such as prohibiting live poultry from remaining overnight and improving the system of inspection and quarantine for live poultry^28^,^42^.

Our study had several limitations. Firstly, it was unfortunate that Wenzhou and Zhoushan in Zhejiang were not covered in our study because some data were missing and there are no LBMs in Zhoushan. Wenzhou is an area with a high incidence of avian influenza, so environmental contamination in LBMs in Zhejiang may have been underestimated because of the lack of data from Wenzhou. Secondly, 85 other poultry-related samples had a high proportion of avian influenza, but sample details were not collected. Fortunately, these samples only accounted for a very small proportion (2.97%, 85/2865) and did not affect our findings. Third, our study was based on cross-sectional evidence, therefore causality could not be deduced and the risk factors for LBM contamination could not be investigated in depth. Therefore, we need further analytic epidemiologic studies for confirmation.

In conclusion, we have demonstrated environmental contamination with A subtype from October, 2015 to March, 2016 in LBMs in Zhejiang province and the risk factors associated with their prevalence in environmental samples, which provide cross-sectional evidence for further research. Drinking water and chopping boards in LBMs were contaminated more severely. The correlation between human infections and H7 prevalence in LBMs during the human influenza season was corroborated. More importantly, we identified risk factors for environmental contamination in LBMs, including the duration of sales per day, types of live poultry, LBM location and the number of live poultry. We recommend that these risks should be targeted to reduce the contamination in LBMs.

## Methods

### Surveillance site selection and sample collection

Environmental surveillance was conducted twice monthly during the epidemic period (from October, 2015 to March, 2016) in Zhejiang province. More than 60 samples were collected per month from at least two kinds of sampling sites including LBMs, poultry rearing farms, backyard poultry, slaughtering and processing plants, habitats for migratory birds and other poultry-related premises. In this study, we selected environmental samples from LBMs in 10 districts. In total, 2865 environmental samples, including 1225 fecal dropping swabs, 615 poultry cage swabs, 224 drinking water samples, 310 sewage samples, 406 chopping board swabs and 85 other poultry-related samples were collected from 292 LBMs. It is important to note that 85 other poultry-related samples, mainly including crib swabs, blood barrel swabs and shedding machine swabs, could not be classified into the above five types.

### Sample transportation, management and laboratory testing

Samples were stored at 4 °C and sent to local network laboratories within 48 hours. The samples were divided into three equal parts and stored in 2-ml screw cap microtubes: the first portion was tested by local network laboratories; the second portion was used for validation by Zhejiang CDC; the last one was transported to the National Influenza Center as a backup. Each portion was at least 1.5 ml and the sample numbers were marked on the screw caps of the microtubes. Within one week after sampling, the sample-related information was entered into the information management system for infectious disease surveillance technology platform and the second and third portions of each sample, stored at −70 °C, were sent to Zhejiang CDC by the network laboratories.

According to the detection method stipulated by the Chinese CDC, each network laboratory conducted nucleic acid testing for avian influenza A virus by real-time quantitative PCR (RT-PCR). If the sample was positive for subtype A, it was typed further for H5, H7 and H9 by PCR. In addition, the network laboratories were required to report A positive testing results to Zhejiang CDC within 24 hours, and Zhejiang CDC subsequently validated the result of the test. However, viruses were not cultured from positive samples to determine whether the viruses were viable. Some of the negative samples were chosen randomly by Zhejiang CDC to test for quality control. All results obtained by Zhejiang CDC were fed back to the corresponding network laboratory. The corresponding network laboratory entered the final test results into the information management system for infectious disease surveillance technology platform. In our study, samples which were positive for H5, H7 or H9 subtypes were defined as “H5/H7/H9 positive”.

### Human cases of infection with H7N9 avian influenza

A clear case definition for use in the diagnosis and treatment protocol for human infections with avian influenza A (H7N9) (2014)^43^ was formulated by the National Health and Family Planning Commission of the People’s Republic of China. Twenty-eight human cases of H7N9 were confirmed in 10 districts of Zhejiang from 1st October, 2015 to 31st March, 2016. The cases of H7N9 were from Hangzhou (11), Ningbo (2), Shaoxing (5), Huzhou (4), Jiaxing (5) and Jinhua (1), respectively. In our study, correlation between human H7N9 cases and environmental contamination with the H7 subtype in LBMs was analyzed to explore the relationship between human infection and LBM contamination.

### Field survey of LBMs

A questionnaire was designed to collect LBM-related information during sampling, which included running days (the number of days the LBM was running for), duration of sales per day, market trading area, quantity of live poultry, the types of live poultry, LBM location, LBM structure, structure of the sales region, rest days, disinfection and cleaning, how live poultry were dealt with, LBM sanitary conditions, sources of live poultry and the management of unsold live poultry. It is important to illustrate that, if a sample was determined to be positive in one LBM, we regarded this market as a positive market; otherwise they were recorded as negative markets.

### Statistical analysis

Epidata (version 3.0, http://www.epidata.dk/) was used for data entry and SPSS 18.0 (SPSS Inc., Chicago, IL, USA) was used for calculation and analysis of the results. The correlation between two variables was analyzed using Pearson’s correlation analysis. Normality was tested using the one-sample Kolmogorov–Smirnov test. The chi-squared test was used to analyze the categorical variables. Rank tests and logistic regression analysis were used to analyze single factors and multiple factors, respectively. All statistical analysis followed the standard that a P value of <0.05 was considered statistically significant. In addition, continuous variables were transformed into categorical variables in the univariate analysis of risk factors and cut-off values for the sub-groups were set as follows: first, “running days” should be no more than 10 days according to government regulations in Zhejiang. So sub-groups of “running days” were set as “0-” and “11-”. Second, in order to explore whether the multiple types of live poultry were risk factors for avian influenza virus, we divided the variable “type of live poultry” into two groups which were “type 1” and “type 2-”(there were more than two types). Third, sub-groups of “market trading area” and “number of live poultry” were divided according to median and quartile range of the variables.

## Additional Information

**How to cite this article**: Wang, X. *et al*. Risk factors for avian influenza virus contamination of live poultry markets in Zhejiang, China during the 2015–2016 human influenza season. *Sci. Rep.* **7**, 42722; doi: 10.1038/srep42722 (2017).

**Publisher's note:** Springer Nature remains neutral with regard to jurisdictional claims in published maps and institutional affiliations.

## Figures and Tables

**Table 1:** Prevalence of avian influenza subtypes in 10 cities.

District	Test result	H5	H7	H9	*x*^2^	*P*
Hangzhou	+	0(0.0)	132(50.0)	164(62.1)	244.609	<0.001
	−	264(100.0)	132(50.0)	100(37.9)		
Ningbo	+	17(10.3)	3(1.8)	59(35.8)	76.756	<0.001
	−	148(89.7)	162(98.2)	106(64.2)		
Shaoxing	+	8(3.8)	14(6.6)	35(16.6)	23.252	<0.001
	−	203(96.2)	197(93.4)	176(83.4)		
Huzhou	+	21(8.0)	55(21.0)	85(32.4)	48.054	<0.001
	−	241(92.0)	207(79.0)	177(67.6)		
Jiaxing	+	6(1.7)	65(18.4)	74(21.0)	65.411	<0.001
	−	347(98.3)	288(81.6)	279(79.0)		
Jinhua	+	8(7.8)	29(28.2)	37(35.9)	23.917	<0.001
	−	95(92.2)	74(71.8)	66(64.1)		
Yiwu	+	0(0.0)	3(4.1)	18(24.3)	29.348	<0.001
	−	74(100.0)	71(95.9)	56(75.7)		
Quzhou	+	56(13.2)	52(12.3)	120(28.3)	46.684	<0.001
	−	368(86.8)	372(87.7)	304(71.7)		
Taizhou	+	14(7.1)	27(13.8)	31(15.8)	7.502	0.023
	−	182(92.9)	169(86.2)	165(84.2)		
Lishui	+	137(16.9)	117(14.4)	243(29.9)	69.523	<0.001
	−	676(83.1)	696(85.6)	570(70.1)		
Total	+	267(9.3)	497(17.3)	866(30.2)	414.771	<0.001
	−	2598(90.7)	2368(82.7)	1999(69.8)		

**Table 2:** Prevalence of avian influenza subtypes in different sample types.

Types	Sum	Positive samples (%)				
		A	H5	H7	H9	H5/H7/H9
Fecal dropping swabs	1225	541 (44.2)	80 (6.5)	159 (13.0)	309 (25.2)	395 (32.2)
Poultry cage swabs	615	321 (52.2)	36 (5.9)	111 (18.0)	190 (30.9)	237 (38.5)
Drinking water samples	224	164 (73.2)	39 (16.5)	46 (20.5)	89 (39.7)	114 (50.9)
Sewage samples	310	187 (60.3)	51 (16.5)	64 (20.6)	94 (30.3)	145 (46.8)
Chopping board swabs	406	255 (62.8)	59 (14.5)	90 (22.2)	145 (35.7)	200 (49.3)
Others	85	51 (60.0)	4 (4.7)	27 (31.8)	39 (45.9)	47 (55.3)
*x*^2^		99.333	64.692	39.351	185.095	71.116
*P*		<0.001	<0.001	<0.001	<0.001	<0.001
Total	2865	1519 (53.0)	267 (9.3)	497 (17.3)	866 (30.2)	1138 (39.7)

**Table 3:** Univariate analysis of risk factors for contamination of LBMs.

Characteristic	N	A			H5			H7			H9		
		Mean rank	*Z/x*^2^	*P*	Mean rank	*Z/x*^2^	*P*	Mean rank	*Z/x*^2^	*P*	Mean rank	*Z/x*^2^	*P*
Running days													
0–	197	128.4	−0.616	0.538	135.49	−2.677	0.007	130.92	−0.386	0.7	129.3	−0.276	0.783
11–	62	135.09			112.55			127.08			132.22		
Duration of sales per day (h)													
0–	102	132.13	−2.075	0.038	140.44	−1.057	0.29	138	−1.298	0.194	141.14	−0.746	0.456
8–	189	153.49			149			150.32			148.62		
Market trading area (m^3^)													
0–	81	129.78	2.553	0.279	123.72	11.983	0.003	137.52	0.811	0.667	131.04	2.08	0.353
25–	130	143.02			141.67			145.32			147.15		
100–	70	150.24			159.75			137.01			141.1		
Number of live poultry													
0–	84	132.63	2.958	0.228	141.17	5.468	0.065	143.17	1.688	0.43	138.53	2.055	0.358
30–	122	147.02			137.23			149.79			141.43		
100–	71	153.87			159.37			135.15			155.66		
Types of live poultry													
1	60	126.44	−1.851	0.064	126.79	−2.305	0.021	128.47	−1.783	0.075	139.43	−0.495	0.621
2–	227	148.64			148.55			148.11			145.21		
LBM location													
urban	102	153.29	−1.09	0.276	147.34	−0.254	0.799	129.19	−2.727	0.006	142.11	−0.598	0.55
rural	189	142.07			145.28			155.07			148.1		
LBM structure													
enclosed	137	140.96	−0.976	0.333	142.07	−0.963	0.335	145.53	−0.099	0.921	143.85	−0.423	0.672
open	154	150.48			149.49			146.42			147.91		
Sales region structure													
Near other region	168	146.88	−0.208	0.835	151.53	−1.674	0.094	142.47	−0.912	0.362	147.72	−0.42	0.675
Independent	123	144.8			138.44			150.83			143.65		
Rest days, disinfection and cleaning													
no	90	136.08	−0.768	0.442	125.08	−2.963	0.003	130.73	−1.66	0.097	138.72	−0.404	0.686
yes	192	144.04			149.2			146.55			142.8		
How live poultry were slaughtered													
Slaughtered	40	150.32	1.076	0.584	143.6	3.388	0.184	131.21	1.37	0.504	142.06	1.746	0.418
Slaughtered in enclosed areas	131	137.7			135.81			142.74			136.88		
Slaughtered in open areas	114	146.52			151.06			147.43			150.36		
LBM sanitary conditions													
poor	45	159.96	7.448	0.024	142.17	0.034	0.983	158.57	6.139	0.046	152.16	3.368	0.186
average	201	145.88			143.95			145.07			145.71		
well	40	113.03			142.76			118.65			122.65		
Live poultry source													
Local areas	196	143.33	0.437	0.804	146.19	1.16	0.56	142.2	1.24	0.538	143.02	4.444	0.108
Other districts in Zhejiang	90	149.73			142.58			151.79			154.15		
Other provinces	4	156.75			177.39			165.88			72.38		
Residual live poultry management													
Slaughtered	9	109.78	3.234	0.199	150.56	0.07	0.966	134.11	1.249	0.535	150.94	0.282	0.869
Stayed in LBMs	170	151.63			144.96			149.65			147.24		
Taken back and fed	111	139.01			145.92			140.07			142.39		

**Table 4:** Logistic regression analysis of risk factors for contamination of LBMs.

Virus subtype	Characteristic	*β*	*S.E*	Wald *x*^2^	*P*	*OR* (95% *CI*)
A	Duration of sales per day	0.132	0.062	4.551	0.033	1.141 (1.011～1.288)
	Types of live poultry					
	1	0.793	0.391	4.104	0.043	2.210 (1.026–4.758)
	2–					
	LBM location					
	Urban	1.026	0.362	8.056	0.005	2.790 (1.374–5.667)
	Rural					
H7	Duration of sales per day	0.143	0.050	8.055	0.005	1.153 (1.045–1.273)
	Number of live poultry	0.002	0.001	4.665	0.031	1.002 (1.000–1.004)
	LBM location					
	Urban	0.708	0.306	5.360	0.021	2.031 (1.115–3.699)
	Rural					
